# A Note on the Appointment of Dental Surgeons to General Hospitals

**Published:** 1902-11

**Authors:** E. Lloyd-Williams


### A NOTE ON THE APPOINTMENT OF DENTAL SURGEONS TO GENERAL HOSPITALS.[1]

BY E. LLOYD-WILLIAMS, M.R.C.S., L.R.C.P., L.D.S.[2]

I AM asked by my American brethren to write a short note on the appointment of dental surgeons to general hospitals, more especially as we, on this side of the water, have had some considerable experience of such appointments.

For the sake of brevity it may suffice to consider two points,—the desirability of having a dentist on the surgical staff of a hospital, and his utility if appointed.

(*a*) Is it advisable to have a dental department of any sort at a general hospital?

Over here we say, Yes! And the fact that every hospital of any note in Great Britain has its dental surgeon goes for something. The intellect is indeed shallow that cannot glean stray wisps of wisdom from history and tradition. The experience of the world is not altogether foolish nor valueless. If the largest city in the world—with the largest hospital system—has persisted for some years in appointing dentists to its hospitals, it may be conceded *a priori* that there must be some possible benefit derived. From a purely selfish professional point of view we are, of course, glad that dentistry should be acknowledged in this way. Our profession is so young that we are apt to forget the modesty which ought to grace youth. To be taken by the hand by our elder sisters, *Medicine* and *Surgery,* and introduced to the circle of healing art is at once a pleasure and an augury of future usefulness. In America oral surgery in its entirety is often in the hands of the dentist, and on this point much might be said for and against; here, the general surgeons are very jealous of any encroachment on their domain, and the matters referred to the dentist by his colleagues are purely dental, with a slight overlapping of such cases as antral abscess, dental cysts, or fractures of the jaws. It is only fair to state, however, that the dentist's advice and assistance are often sought in numerous operations about the mouth, where his special

---

[1] Read before The New York Institute of Stomatology, May 6, 1902.

[2] Dental Surgeon to the Dental Hospital, London.

dental knowledge is likely to be of value. From this we may gather that the staff of a hospital considers that it derives assistance from a dental colleague. The question as to the benefit to the public is pertinent to our second inquiry:

(*b*) Is the dentist of real use? Undoubtedly to his colleagues and in special cases; but, generally speaking, does the suffering *clientele* of the hospital benefit?

Let us inquire as to the duties which it is possible for him to perform. First of all, he can advise; and good advice may mean much to a sufferer. Secondly, he can give the more immediate relief of extraction. Some pious dentists hold up their hands in horror at the very mention of the word! What is the poor woman, who has left six children at home in the charge of a neighbor, to do with a raging attack of pain from an abscessed molar? "Pay several visits to a dental hospital and try and have the tooth saved," say some of the smug exponents of a pseudo-conservative turn of mind. In hundreds of instances the very poor *cannot* leave their work for prolonged treatment. It is very sad; but we cannot revolutionize the social conditions of the poor in dental matters all at once, and it still remains a fact that in very many cases the only possible relief of dental suffering lies in cold steel. Very often the patient is anxious and willing to have treatment; and here the difficulty arises that one man cannot possibly devote sufficient time in order to cope with a large number of cases. At some of our larger hospitals it is possible to find dressers from the medical students who are anxious to carry out treatment of an elementary nature, and many dressings, temporary fillings, etc., are done. This, however, is the exception; and it stands to reason that a man, single-handed, can do but little conservative work. But what he can do is this, and here, in my opinion, lies one of the principal benefits of the appointment: he can advise treatment, explain its nature and importance, and influence the patient to apply at a special dental hospital, where every facility is at his service. This is largely done in London hospitals, and the real benefit is perhaps larger than might at first sight appear. For these reasons, hurriedly expressed, I advocate strongly the appointment of dental surgeons to general hospitals; the result is not perfect, but it does some amount of good. I once heard the question propounded, "Is marriage a failure?" with the reply, "Yes; but it's the best we've got!"

